# Adaptive Data Transmission Algorithm for the System of Inertial Sensors for Hand Movement Acquisition

**DOI:** 10.3390/s22249866

**Published:** 2022-12-15

**Authors:** Michał Pielka, Paweł Janik, Małgorzata A. Janik, Zygmunt Wróbel

**Affiliations:** Faculty of Science and Technology, Institute of Biomedical Engineering, University of Silesia in Katowice, ul. Będzińska 39, 41-200 Sosnowiec, Poland

**Keywords:** transmission control algorithm, wireless sensor, wearable system, hand MoCap, MEMS, IoT

## Abstract

Modern systems of intelligent sensors commonly use radio data transmission. Hand movement acquisition with the use of inertial sensors requires the processing and transmission of a relatively large amount of data, which may be associated with a significant load on the network structure. Network traffic limitation, without losing the quality of monitoring parameters from the sensor system, is therefore important for the functioning of the radio network which integrates both the teletransmission sensor system and the data acquisition server. The paper presents a wearable solution for hand movement acquisition, which uses data transmission in the Wi-Fi standard and contains 16 MEMS (Micro Electro Mechanical System) sensors. An adaptive algorithm to control radio data transmission for the sensor system has been proposed. The algorithm implemented in the embedded system controls the change of the frame length, the length of the transmission frame and the frequency of its sending, which reduces the load on the network router. The use of the algorithm makes it possible to reduce the power consumption by the sensor system by up to 19.9% and to limit the number of data transferred by up to about 91.6%, without losing the quality of the monitored signal. The data analysis showed no statistically significant differences (*p* > 0.05) between the signal reconstructed from the complete data and processed by the algorithm.

## 1. Introduction

Gesture recognition systems using IMU (Inertial Measurement Unit) sensors are solutions in which relatively large amounts of data are processed. Thus, the efficiency of such systems is related to the methods of processing data from sensors and their transmission. Wearable solutions for monitoring movement or other body parameters use radio data transmission [1,2,3,4], including transmission in the ISM (Industrial, Scientific, Medical) 2.4 GHz band. Within this band, radio transmission is implemented in many standards, such as Wi-Fi (IEEE 802.11) [5,6], Bluetooth (IEEE 802.15.1) [7,8] or ZigBee (IEEE 802.15.4) [9,10]. Here, it is important to point out the key problems related to the processing and radio transmission of data in sensor networks. Devices using radio transmission in these technologies may be in close proximity and interfere with each other [11]. Moreover, the use of a large number of wireless IMU systems can lead to their mutual interference. This causes a decrease in the efficiency and reliability of transmission, which reduces the functionality of these systems. For this reason, it is important to reduce radio band occupancy, for example, by limiting radio transmission. Radio transmission also has a significant impact on the energy demand of sensor systems, therefore, limiting the use of teletransmission modules allows for a reduction in electricity consumption. In turn, reducing energy demand is of particular importance in the case of solutions using battery power supply. There are many different methods of radio transmission control to optimize network traffic or reduce energy consumption. The latter can be achieved by monitoring the network and controlling the frequency of connection events (mainly in Bluetooth Low Energy), transmission speed, and transmitter power [12,13]. In turn, the transmitter power can be controlled based on the input data from the system sensors [14]. Transmission control using the input data can be implemented using machine learning [14]. One of the methods for reducing teletransmission band occupancy is data aggregation. In the Wi-Fi standard (802.11), a mechanism for sending data blocks containing several data frames has been introduced, wherein the receipt of the data block is acknowledged by one ACK (Acknowledgment). Controlling the length of data blocks can be used to optimize network traffic while maintaining transmission reliability [15]. The network radio traffic can also be limited by dividing the network into clusters with a master node as well as assigning different priorities to data and data queuing [16]. Solutions modifying MACs in terms of prioritizing data are also proposed [17], and time slots of variable length [18] or dedicated slots for critical data transmission are introduced [19]. Modifications of the MAC layer require appropriate implementation in all cooperating network devices. Optimization of radio network management is also carried out by modifying the routing protocols by introducing specialized supervision devices [20]. Such solutions involve sending additional information about the packet path and implementing algorithms, e.g., ant colony [21] or fuzzy logic [22]. In the case of coexistence of several networks with many access points, optimization methods are introduced that make it possible to select an access point for the network device, which is used, for example, in cellular networks [23]. Reducing the network load may also be realized by using two networks operating in separate bands in parallel (for example Wi-Fi and LTE/3G) [24,25]. Radio data transmission can also be optimized by limiting the data packet length, for example, by sending only differences between consecutive values, which may limit the scope of sent data and allow the original data to be restored [26]. In order to reduce the energy expended on radio transmission and limit the amount of transmitted data, the measurement signal sampling frequency is controlled in sensor systems. For this purpose, algorithms are used that limit signal sampling depending on the nature of the input data [27,28,29]. However, limiting the amount of data transferred may lead to the loss of some data [30]. Signal sampling is also controlled through the use of dedicated electronic systems that allow for the detection of a specific nature of monitored signals [31]. Effective management of radio data transmission is important in the sensor systems for motion acquisition, which allows to reduce the energy demand of electronic systems and reduce network traffic [32,33]. One of the methods of radio transmission optimization in the case of generating a large amount of data by sensors is, for example, the selection of a minimum data set necessary to describe the monitored process [34].

The article presents a new transmission protocol for the sensor network in the form of an algorithm designed both to increase the energy efficiency of the radio link and to reduce the load on the Wi-Fi sensor network. This approach to radio data transmission management seems to be universal, as the mentioned aspects are important for the practical implementation of the developed algorithm. The proposed method allows for transmission control only on the basis of data from sensors. In addition, the transmission process is controlled without the need for two-way communication between the sensor system and another device supervising the transmission or routing process (e.g., PC server). The developed transmission method does not require the transmission of additional control frames, which reduces the network load. Furthermore, the transmission control algorithm does not modify the 802.11 MAC layer, nor does it introduce additional routing protocols, which simplifies its implementation, in particular in sensor networks with limited hardware resources. Dedicated electronic systems are also not required to control the transmission. Therefore, the proposed algorithm can be used in sensor network nodes with a reduced architecture [35,36,37]. Moreover, a constant sampling frequency is maintained for all MEMS sensors. The transmitted data do not require additional processing on the part of the receiving device.

The article is divided into three main parts. Section 2 and Section 3 describe the measuring stations used in the research cycle as well as the hardware and logical architecture of the developed wearable system with a dedicated transmission control algorithm. Section 4 concerns testing the presented hardware solution and the algorithm in terms of energy efficiency and load of the sensor network as well as the quality of traffic mapping. Section 5 is related to the analysis and discussion of the obtained results.

## 2. Measurement Setup

### 2.1. Measuring Stations

**Figure 1:**
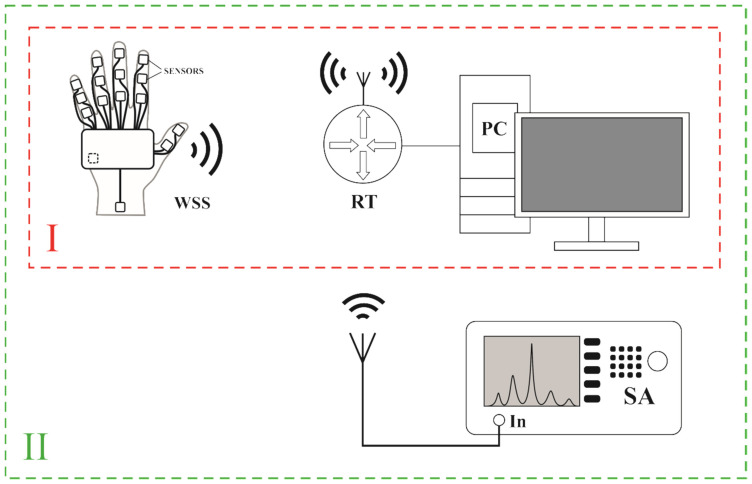
Station for measuring the network band occupancy.

Three measuring stations were designed to test the adaptive data transmission algorithm developed for a system of inertial sensors. Figure 1 presents the structure of two measuring stations. The first station is marked with the Roman number I. It contains the WSS (Wearable Sensor System) described in detail in Section 2.2, router (RT) with an access point (RT-N10U) and PC server. The WSS radio transmits data from sensors in the Wi-Fi 2.4 GHz standard. The frames transmitted by the WSS were filled with data from the sensors, and the value of the transmission filter control parameter was simulated, which is described in Section 3. In turn, the RT receives Wi-Fi frames containing data transmitted by the WSS, and then the RT via a wire (cable with RJ45 plug) sends Ethernet frames using UDP (User Datagram Protocol) to the PC server, where the data are archived.

The station marked with II (Figure 1) was designed to test the network load in the context of radio band occupancy. Station II is an extended version of the previously described Station I. Station II additionally includes SA—(spectrum analyser RSA5065-TG), which allows for density spectrogram registration. The AARONIA HyperLOG 7060 antenna was attached to RSA5065-TG.

**Figure 2:**
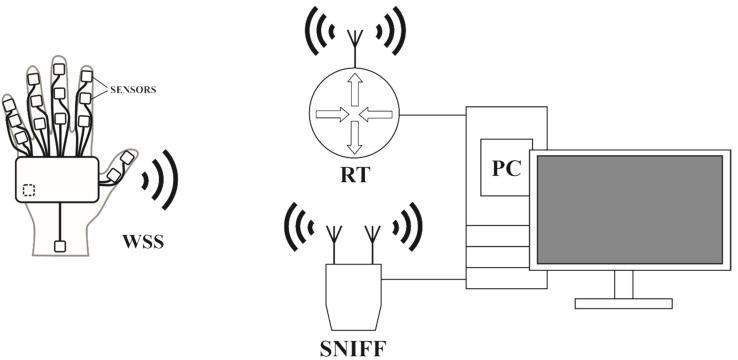
Station for measuring the load on the sensor network.

Figure 2 presents the station for the analysis of network traffic generated by the WSS. Like in the case of the stations I and II, the RT allows for the registration of only data frames (Ethernet), whereas the network traffic is monitored in a wider range via the sniffer–ALFA AWUS036ACH AC1200. The sniffer connected to the PC server via the USB port was used to receive Wi-Fi frames. The measuring station makes it possible to monitor the simulated load on the radio network with a different amount of transmitted data, which is described in more detail in Section 4.

In the stations presented in Figure 1 and Figure 2, the network created by the WSS, the RT with an access point and the PC server use the IPv4 protocol. The Beacon time (packets sent by the access point to synchronize a wireless network) of the RT was 100 ms, the DTIM (Delivery Traffic Indication Message) value was 3. The Wi-Fi access point channel was selected automatically. The data from the WSS module are sent to the PC server using the UDP. The data section of the UDP frame in the context of the adaptive transmission algorithm is discussed in more detail later in the article.

### 2.2. Wearable Sensor System

**Figure 3:**
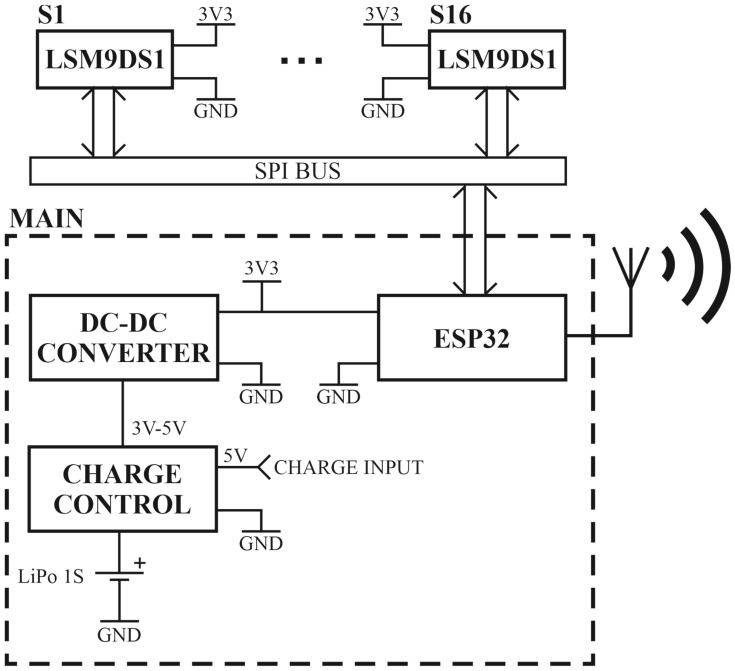
Block diagram of the wearable sensor system (WSS).

**Figure 4:**
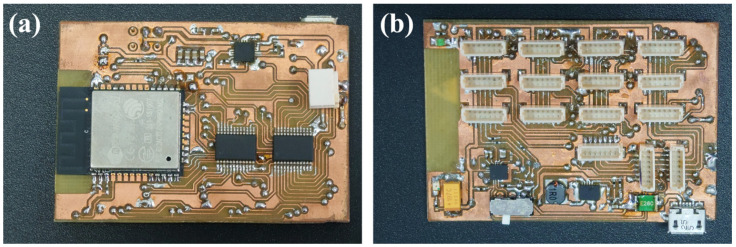
View of WSS: (**a**) from the Wi-Fi module side, (**b**) from the connectors side.

The developed sensor system for hand motion acquisition is presented in the form of a block diagram in Figure 3. Hand motion acquisition with the use of inertial sensors is related to the implementation of such a system in a wearable form. The solution presented in the article consists of a central module (MAIN), to which sixteen LSM9DS1 sensors [38] (S1 to S16) are connected via the SPI (Serial Peripheral Interface) bus (SPI BUS). Each of the sensors has a built-in accelerometer, gyroscope, and magnetometer. The sensor system is powered by a 3.7 V lithium polymer battery (1 s). The battery is connected to the CHARGE CONTROL system, which, while charging the battery, is powered from an external 5 V source connected to the CHARGE INPUT. The CHARGE CONTROL output is connected to the DC-DC CONVERTER input. The converter output voltage is 3.3 V and is used to power both the ESP32 module and the LSM9DS1 sensors. ESP32 [39] is a single 2.4 GHz Wi-Fi-and-Bluetooth combo chip, which in the presented wearable solution downloads data from the LSM9DS1 sensors, processes the received data and transmits them to the computer. The main board of the central module (MAIN) is shown in Figure 4a,b.

**Figure 5:**
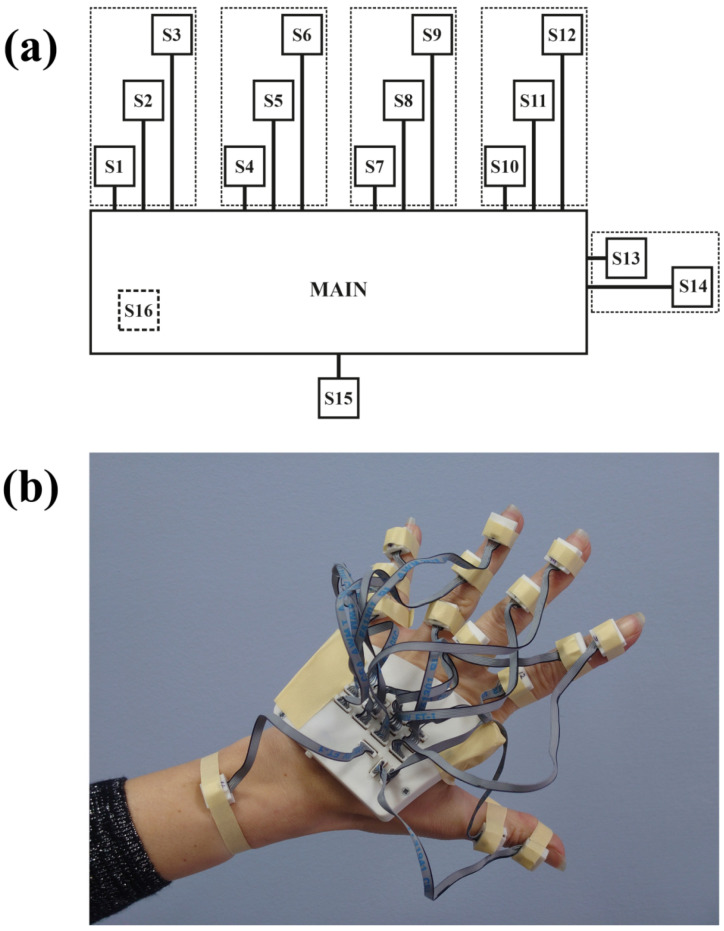
Arrangement of sensors in WSS: (**a**) block diagram and (**b**) view of the system placed on the hand.

The location of the individual sensors of the hand motion acquisition system is presented in Figure 5a,b. Sensors S1–S15 (external) are connected to the MAIN module via multi-core cables, whereas the S16 (internal) sensor is integrated with the main board of the MAIN module. The S15 sensor is placed on the forearm, whereas each of the other external sensors (S1 to S14) is placed on the corresponding phalanx of individual fingers (Figure 5). There are two sensors for the thumb, whereas for the other fingers, three sensors are implemented in the system. The applied location of the sensors allows both to monitor the movement of each of the fingers as well as the position of the hand in relation to the forearm.

## 3. Adaptive Data Transmission Algorithm

**Figure 6:**
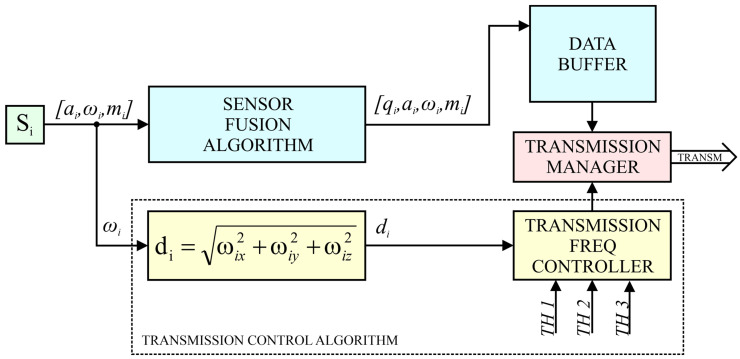
Logical diagram of WSS operation.

**Figure 7:**
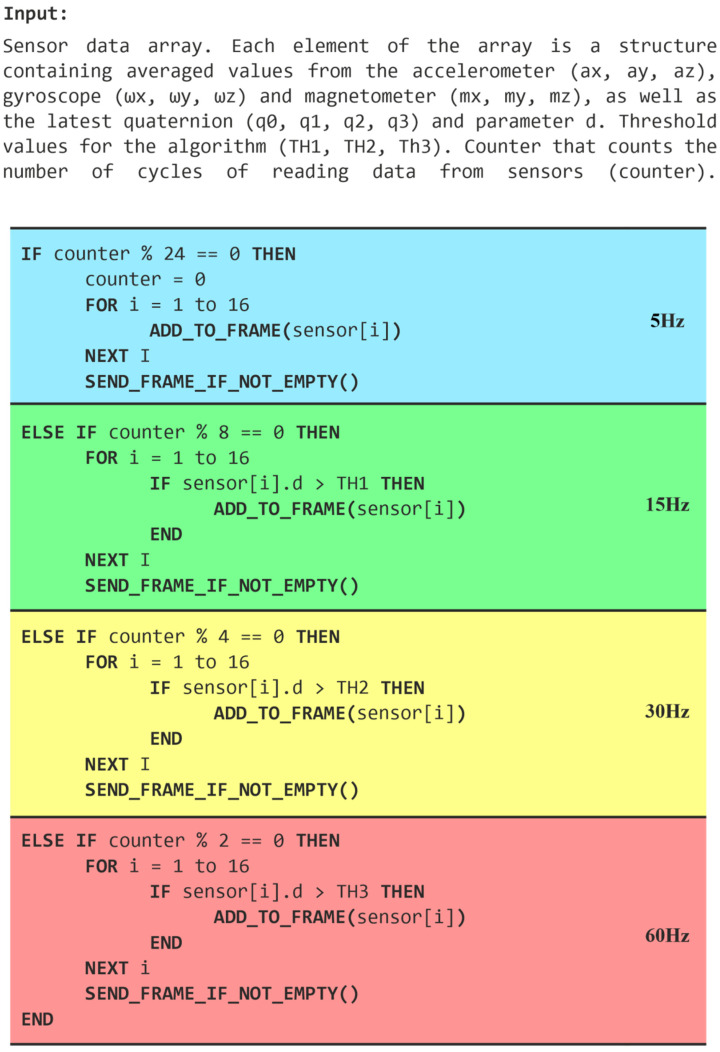
Pseudocode for the transmission control algorithm.

The wearable sensor system is also an embedded system with the implemented adaptive data transmission algorithm. The general structure of the WSS operation is presented in Figure 6, where the block of the radio transmission control algorithm is marked with a dashed line. This algorithm allows to increase the energy efficiency of the WSS and reduce the load on the sensor network. In turn, the input data of the algorithm are the vector $\omega_{i}$ and the defined threshold values TH1, TH2, and TH3. In addition, the WSS operation diagram includes the processing of vectors $a_{i}$, $m_{i}$, and the quaternion $q_{i}$, which is also indicated in the pseudocode in Figure 7. During operation, the ESP32 module collects data from S*_i_* sensors (*i* = 1, 2,…, 16). The sampling frequency of sensors in the implemented system for the accelerometer and the gyroscope is 119 Hz. The data obtained from each sensor are in the form of three vectors $a_{i}$, $\omega_{i}$, $m_{i}$ (1), where *i **=*** 1, 2, …, 16 denotes the indexing of the sensor number.

(1) $$a_{i}=\left[a_{ix},a_{iy},a_{iz}\right]\;\;\;\omega_{i}=\left[\omega_{ix},\omega_{iy},\omega_{iz}\right]\;\;\;m_{i}=\left[m_{ix},m_{iy},m_{iz}\right]$$

The vectors $a_{i}$, $\omega_{i}$, $m_{i}$ represent the data of individual sensors. The coordinates $a_{ix}$, $a_{iy}$ and $a_{iz}$ of the vector $a_{i}$ correspond to the acceleration in relation to the x, y i z axes, respectively. Similarly, the coordinates $\omega_{ix}$, $\omega_{iy}$, $\omega_{iz}$ correspond to the rotational speeds of rotations performed by individual sensors around the x, y and z axes. In turn, the coordinates $m_{ix}$, $m_{iy}$, $m_{iz}$ correspond to the values of the magnetic field induction measured by the magnetometers of the individual sensors.

The data obtained from the *i*-th sensor are used by the fusion algorithm (SENSOR FUSION ALGORITHM) to determine the quaternion $q_{i}$. An algorithm for AHRS (Attitude Heading Reference System) was used for the sensor Fusion [40]. The data ($a_{i}$, $\omega_{i}$, $m_{i}$) from the sensor and the quaternion $q_{i}$ are saved in the data buffer along with the index number of the *i*-th sensor to which they correspond.

The operation of the data transmission control algorithm requires only a small number of simple arithmetic operations. The results of testing the algorithm in the context of energy efficiency are presented in more detail in Section 4.2. In turn, Section 4.3.1 and Section 4.3.2 show the algorithm effectiveness in terms of optimizing network traffic.

On the basis of the rotational speed vector $\omega_{i}$, the parameter $d_{i}$ is also determined according to the Formula (2) and is related to the resultant angle of rotation of individual sensors. Taking into account the rotation from all system axes allows for more effective transmission.

(2) $$d_{i}=\sqrt{\omega_{ix}{}^{2}+\omega_{iy}{}^{2}+\omega_{iz}{}^{2}}$$

The parameter $d_{i}$ calculated for the *i*-th sensor is used to control the radio transmission (TRANSMISSION FREQ CONTROLLER). Based on the values of parameters $d_{1}$–$d_{16}$, the frequency of radio data sending from individual sensors and the content of individual transmission frames are determined. Four frame transmission frequencies *f_T_* were defined in the transmission control algorithm (Figure 6): 60 Hz, 30 Hz, 15 Hz and 5 Hz. The *f_T_* frequencies are approximate values and result from dividing the sensor sampling frequency (119 Hz) by the numbers 2, 4, 8 and 24, respectively.

The input data of the algorithm are also three threshold values TH1, TH2, and TH3, fulfilling the condition: 0<TH1<TH2<TH3. Based on the result of comparing the value of the parameter $d_{i}$ with the threshold values *TH*1, *TH*2 and *TH*3, the frequency of data transmission *f_T_* from the *i*-th sensor is determined (Table 1).

The individual threshold values *TH* were determined on the basis of preliminary analyses, which allowed for assigning the speed of motion to four ranges. The slowest movements are associated with a threshold below *TH*1, whereas the fastest with a threshold above *TH*3. Defining the threshold values *TH* is related to the dynamics of the monitored traffic. Theoretically, too low threshold values of the algorithm will increase the frequency of frame transmission even with slow movements. In this case, the effectiveness of network traffic reduction will decrease. On the other hand, too high threshold values will negatively affect the smoothness of motion mapping. When monitoring movements, the system will tend to transmit frames at lower frequencies, which may result in significant delays in movement mapping. In this case, only the most dynamic movements will activate the increase in frame transmission frequency, and therefore only they will be mapped without delays on the server side. The procedure for determining the threshold values is described in more detail in Section 4.3.3.

By correlating the frequency of sending frames with the threshold values, it is possible to optimize radio network traffic, which allows for the reduction of bandwidth usage and more effective management of the energy expended on data transmission. Limiting only the transmission frequency and aggregation of data in a frame (frame lengthening) will limit network traffic. However, in the case of monitoring fast movements, it will not ensure their smooth (real-time) mapping. The lack of smoothness in mapping fast movements is related to the relatively long interval of transmission of successive data portions (e.g., 200 ms). Data aggregation consists in placing the measurement points obtained from the sensors from the moment of sending a previous data frame in the teletransmission frame, as discussed in previous publications [25,26]. However, in the case of the adaptive algorithm presented in the article, the data were not aggregated due to the large number of sensors (16 sensors). Teletransmission frames contain the current quaternion $q_{i}$ and the averaged (arithmetic mean) values of the vectors $a_{i}$, $\omega_{i}$, and $m_{i}$, obtained from one or more sensors. All values derived from a specific sensor since the moment of sending a previous data frame are averaged.

The algorithm is presented in the form of a block pseudocode (Figure 7), in which the sections responsible for data transmission with declared frequencies are grouped graphically.

**Figure 8:**
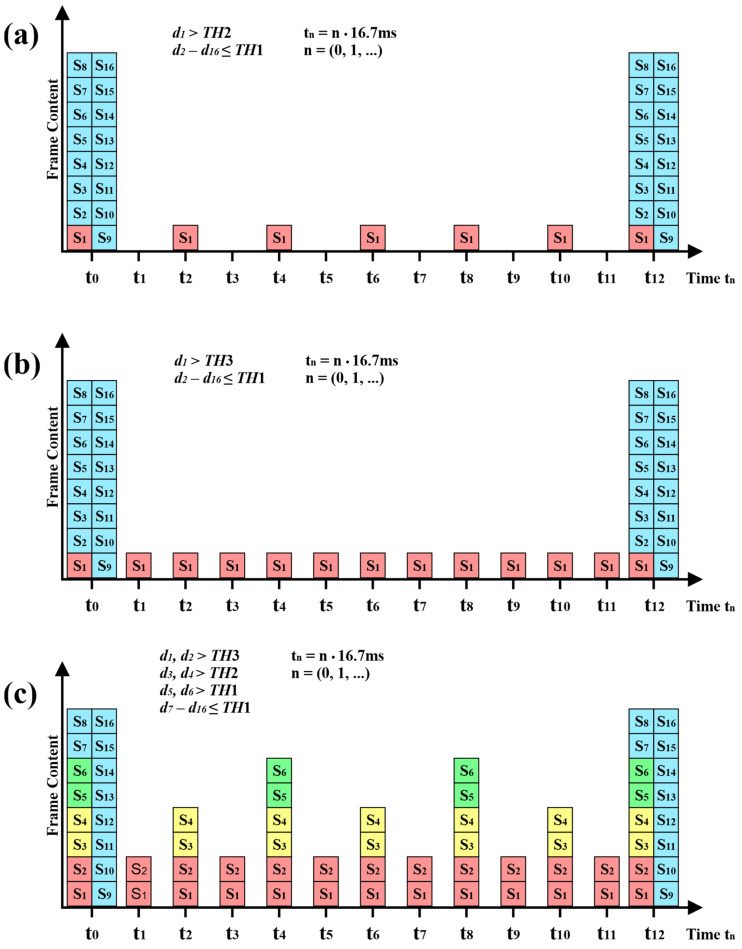
Structure of data transmission from individual MEMS sensors.

Data from all sensors are stored in the data buffer and then placed in common data frames (the data structure is presented in Appendix A) and transmitted with a frequency of 5 Hz. Depending on the value of the parameter $d_{i}$, data from individual sensors can be transmitted at several frequencies, which is associated with sending additional frames. Figure 8 presents examples of data transmission structures from individual sensors, with the transmission frequencies *f_T_* being presented in the form of time slots with an interval of 16.7 ms. If the value of the parameter $d_{i}$ for at least one sensor exceeds the TH1 threshold, then the data transmission for these sensors is performed with a frequency *f_T_* of 5 Hz and additionally 15 Hz. On the other hand, when the value of the parameter $d_{i}$ for at least one sensor exceeds the TH2 threshold, then the data transmission for these sensors is carried out at frequencies *f_T_* of 5 Hz, 15 Hz and additionally 30 Hz, which is presented graphically in Figure 8a. The situation is similar in the case of exceeding the TH3 value, when the frames are additionally transmitted with a frequency of 60 Hz (Figure 8b). Figure 8c presents an example of a frame transmission structure in which different frequencies *f_T_* (5 Hz, 15 Hz, 30 Hz, and 60 Hz) are used to transmit data from different groups of sensors.

## 4. Measurements and Testing of the Measuring System

### 4.1. Measurement Protocol

The developed adaptive data transmission algorithm was tested in terms of energy efficiency and network load. For this purpose, a measurement protocol was designed, which consisted of 26 measurement cases, P1 to P26. The measurement protocol controlled the data transmission from 16 sensors, which allowed to simulate the practical application of the algorithm. In individual measurement cases, data from sensors were transmitted with predefined frequencies *f_T_*. The tested measurement cases are presented in Table 2. The measurement protocol includes data transmission from all 16 sensors both at the highest frequency *f_T_* of 60 Hz (P1) and the lowest frequency of 5 Hz (P26). The cases from P2 to P25 represent selected intermediate transmission states of the sensor network.

For example, in the measurement case P21, the data are transmitted for 15 sensors with the frequency *f_T_* equal to 5 Hz, and for one sensor with the frequency *f_T_* equal to 5 Hz, 15 Hz, 30 Hz, and 60 Hz (for the sensor, for which data are transmitted at a given frequency *f_T_*, data are also transmitted with all available frequencies below *f_T_*). Measurements of each case from P1 to P26 were recorded for 20 s. During this time, the MEMS sensors made continuous measurements, and their parameters $d_{i}$ (the parameter controlling the transmission filter) were set in such a way as to ensure the frequency of data transmission *f_T_* from individual sensors in accordance with the developed measurement cases.

### 4.2. System Current Consumption Testing

The energy efficiency tests of the system with the implemented adaptive algorithm were carried out on the basis of current consumption. The supply voltage of the system was 4 V. The current consumption was determined by measuring the voltage across the shunt resistor. Due to slight differences between the measured current consumption values (below 0.5%), the measurement cases were grouped in accordance with Table 3.

All measurement cases where the maximum frequency *f_T_* was 15 Hz (P2, P8, P14, P19, and P24) are characterized by similar current consumption (about 242 mA). A similar value of the consumed current (about 251 mA) was recorded in the tested measurement cases, where the maximum frequency *f_T_* was 30 Hz or 60 Hz.

The tests, conducted in accordance with the measurement protocol, show that the greatest savings in the current consumed by the system are obtained when data from sensors are sent with the frequency *f_T_* equal to 5 Hz. A reduction in the system current consumption in this case can even reach 19.9% in relation to the system in which data from sensors are transmitted at frequencies of 60 Hz or 30 Hz.

### 4.3. Network Load Testing 

#### 4.3.1. Measurements of Radio Band Occupancy

**Figure 9:**
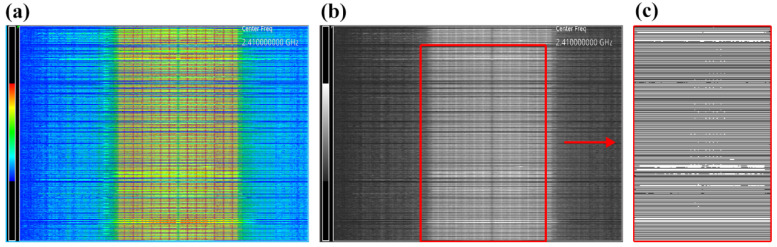
Stages of spectrogram image analysis: (**a**) input image, (**b**) grayscale image, (**c**) image after thresholding.

The network load status test was carried out both in terms of radio band occupancy and data transmission. During the performed tests, the WSS transmitted data in accordance with the previously developed measurement protocol. Measurements of radio band occupancy depending on the frame transmission frequency f_T_ and the length of transmission frames were carried out using the measuring station presented in Figure 1. The spectrum analyser (SA) performed the measurements in the RTSA (Real-Time Spectrum Analyser) mode and displayed spectrograms. The measurements were performed in the frequency range from 2.39 GHz to 2.43 GHz. The WSS sensor module communicated with the RT access point using channel 1 (2.412 GHz). The acquisition time of one measurement was set to 24.996 ms. The measurement antenna directed at the WSS was 1 m away. The band occupancy tests, depending on the test case, were performed on the basis of the analysis of recorded spectrograms, where the X axis represents the frequency, and the Y axis represents the time. The Y axis consists of 483 points, with each point representing a single SA analyser measurement (24.996 ms). The signal strength measurement range represented by the spectrogram colours is from −100 dBm to 0 dBm. However, it was limited and ranged from −72 dBm to −27 dBm (Ref Hue Pos was set to 73 and Bottom Hue Pos to 28). Measurements were taken for all the test cases from P1 to P26 and, additionally, a measurement was performed with the sensor module turned off to determine the effect of ambient noise on the other measurements. The measurement results were saved as bitmaps presenting spectrograms. For the purposes of further analysis, the spectrogram colours were changed. The default colour range (Figure 9a) was converted to grayscale from the hexadecimal value range 0xFFFFFF (white) to 0x242424 (dark grey), where white is the highest measured signal strength (Figure 9b). From each image, the central part of the monitored band with a range of about 18 MHz was selected. Then, each image was thresholded with the values of 75, 100, 125, and 150 (from the range of 0–255, where 0 is black, 255 is white).

**Figure 10:**
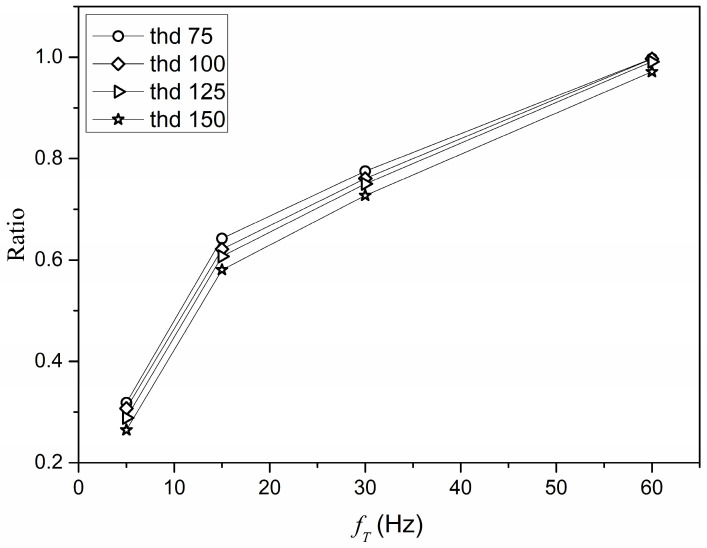
Graph of the proportion of white in the images depending on the frequency *f_T_*.

For each of the processed images (Figure 9c), the percentage of white was calculated, which represents the band occupancy. Regardless of the adopted threshold value, the share of white in the background image does not exceed 2.2%, so it can be concluded that the background noise did not have a significant impact on the measurement results. Figure 10 shows a graph of the percentage of white in the images as a function of the frequency *f_T_*, for various threshold values (thd).

For individual threshold values (75, 100, 125, and 150) and measurement cases with the same maximum frame transmission frequency *f_T_*, the obtained values of the percentage of white are similar, because the difference between the mean and the individual percentage values does not exceed 3.7%. For this reason, cases with the same maximum frame rate were analysed as being equivalent. Moreover, irrespective of the threshold value, the curves of the percentage of white for the measurement cases with the maximum frequency *f_T_* are of a similar nature (Figure 10). Table 4 shows the mean percentage reduction in the band occupancy for transmitting frames at 5 Hz, 15 Hz, and 30 Hz, relative to transmission at 60 Hz, calculated using all curves.

#### 4.3.2. Transmission

**Figure 11:**
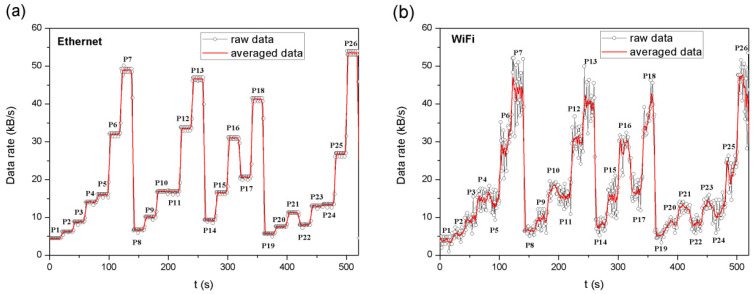
Changes in data rate for defined measurement cases: (**a**) for Ethernet frames and (**b**) for Wi-Fi frames.

To test the network load in terms of the amount of transmitted data, the measuring station presented in Figure 2 was used. The WSS module transmitted data in accordance with the developed measurement procedure (26 measurement cases). During the system operation, Ethernet transmission frames, which use the UDP, were acquired on the PC server side using Wireshark. Then, the Wi-Fi frames captured by the Sniffer were also acquired. Only the data frames sent by the WSS were analysed. In the measurement cases (P1, P2, P8, P14, P19, and P24), for which the maximum frame transmission frequency *f_T_* was 5 Hz or 15 Hz, Management frames were captured informing that the radio module goes to sleep or is woken up. These frames were not observed for the other measurement cases. Putting the radio module into the sleep mode allows to reduce power consumption, which was observed while testing the system energy efficiency (Section 4.2). In addition, the amount of data transferred was calculated (in kB/s), in which the Management and ACK frames captured by the Sniffer were omitted. The mean data rate during 1 s was calculated for both Ethernet and Wi-Fi frames. The graph of the amount of transmitted data versus time, taking into account the measurement cases (P1 to P26), is shown in Figure 11.

In the case of Ethernet frames, the data include: data from the system, UDP, IP, and MAC 802.3 headers, whereas in the case of Wi-Fi frames, the total length of the frame sent by radio (including the radio interface configuration header, WPA2, MAC 802.11, LLC, SNAP, IP, and UDP). Due to the greater number of components and the fact that the 802.11 MAC header is longer than the 802.3 MAC, the total length of the Wi-Fi frame will be greater than for the Ethernet frame. Attention should be drawn to the difference in the amount of data captured by the Sniffer compared to the Ethernet transmission. The amount of data from the captured Wi-Fi frames should be greater than the amount of data from the Ethernet frames. In some measurement cases, the opposite is true because the Sniffer cyclically switches between Wi-Fi channels, which leads to partial data loss, but the shape of the curve remains intact.

Reducing the maximum frequency with which data frames are transmitted, and possibly increasing the number of sensors for which data frames are transmitted at a frequency *f_T_* of 5 Hz, allows for a significant reduction in the amount of data transmitted. This reduction does not have a significant impact on the quality of motion mapping on the PC server side, which is discussed later in the article. Most data are sent in the case of P26, whereas the least in P1. In the case of P1, the amount of data transferred is by approximately 91.6% lower than in the case of P26. The percentage differences between P26 and the other cases are presented in Table 5.

#### 4.3.3. Quality of Motion Mapping Using the Data Transmission Algorithm

In the presented system, when optimizing network traffic, it is important to reduce the amount of transmitted data, while maintaining high-quality monitoring of individual sensor signals. In the algorithm described in Section 3, data from the accelerometer, gyroscope and magnetometer are averaged and then sent to the PC server. Additionally, the latest quaternion representing the rotation of the individual sensors is sent. This reduces the number of measurement points depending on the frame transmission frequency *f_T_*. In order to verify the quality of mapping the measurement signals, a series of tests was carried out with the use of the proposed algorithm. The research allowed to compare the reduced data received from the WSS and the complete measurement data from the MEMS sensors. The tests were preceded by the procedure of determining the threshold values (*TH*1, *TH*2, *TH*3).

**Figure 12:**
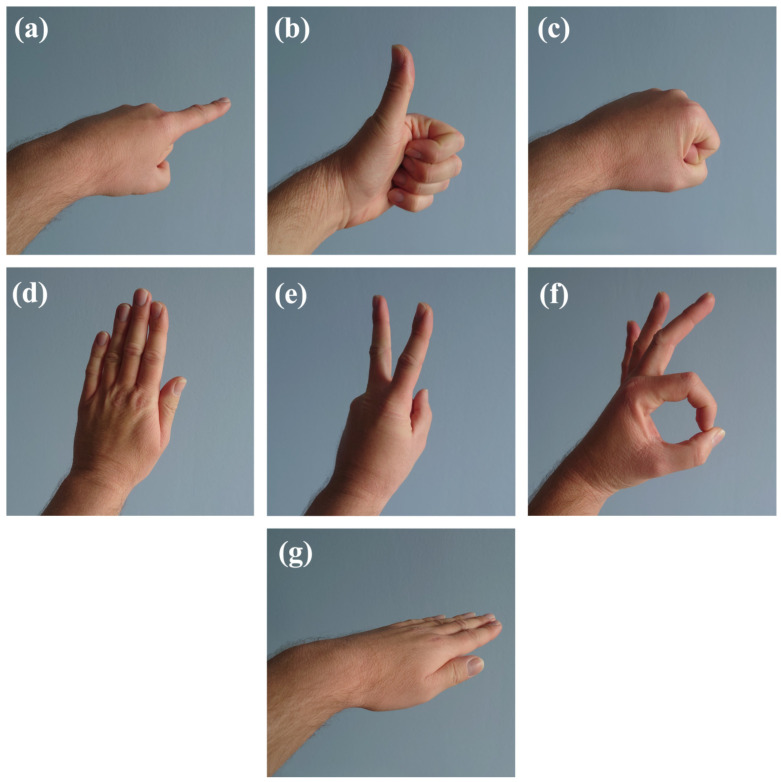
View of the hand when performing gestures: (**a**–**f**) defined measurement gestures and (**g**) control gesture.

In order to determine the threshold values, measurements were performed with the participation of three volunteers. During the measurement, the WSS transmitted all measurement data from the MEMS sensors (the transmission control algorithm was disabled). Each volunteer performed six measurement gestures, which are presented in Figure 12a through Figure 12f. For the purposes of the research, a measurement procedure was designed, in which the time of performing individual gestures and the time of the control gesture were determined. The set times ensured repeatability in reproducing the sequence of gestures by individual volunteers. In addition, the performance of successive gestures in accordance with the adopted measurement procedure ensured the diversified hand movement dynamics as well as the diversification of the input data for testing the transmission algorithm. Each measurement gesture was performed for 3 s. In the measurement procedure, a control gesture was also introduced—the hand was positioned horizontally with the fingers extended (Figure 12g). The control gesture was performed before the beginning of the series of measurement gestures, after its completion and between each of the measurement gestures. The control gesture was also performed for about 2–3 s. With the participation of volunteers, three measurement cycles were carried out, in which three series of gestures were performed (9 series in total). There was a 10-s pause between the series of gestures.

From the received data, only the values from the 3D gyroscopes were analysed, and the $d_{i}$ parameter was calculated for each vector $\omega_{i}$ according to the Formula (2). Then, the local maxima were determined for the data from all the series of measurements. The maxima occurred at the time of performing the measurement gesture, or switching to the control gesture (12 maxima per series). The arithmetic mean was calculated from the set of maxima. The threshold values of *TH*1, *TH*2, and *TH*3 were ¼, ½, and ¾ of the calculated mean, i.e., 84.21, 168.42, and 252.63, respectively.

## 5. Results and Discussion

### 5.1. Acquisition of Data from Sensors

The WSS, with the implemented control algorithm, transmitted two types of frames: frames containing complete data from the MEMS sensors sent with a frequency of 119 Hz and frames modified by the algorithm. The study of the hand motion mapping quality based on data modified by the transmission control algorithm was carried out on the basis of measurements with one volunteer. A series of measurements was performed, consisting of the previously described three repetitions of a predetermined series of gestures. Then, the data obtained from the frames created by the algorithm were compared with the complete data from the MEMS sensors.

**Figure 13:**
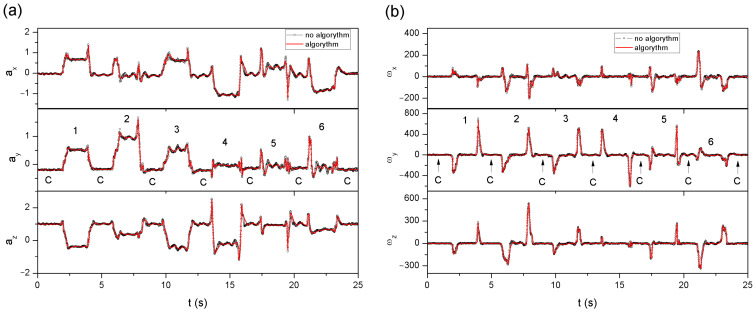
The first measurement series, consisting of six gestures (1–6) separated by the control gesture C, recorded from the sensor No. 8: (**a**) change in the values of individual components obtained from the accelerometer, (**b**) change in the values of individual components obtained from the gyroscope.

Examples of measurement signals (from complete data and after applying the algorithm) from one series of gestures, obtained from the accelerometer and the gyroscope, are presented in Figure 13a,b, respectively. The presence of subsequent gestures 1–6 (Figure 12a–f) and the control gesture C (Figure 12g), visible as a flattened signal between individual gestures (Figure 13), have been marked.

It can be observed in Figure 13 that the use of the algorithm does not significantly change the nature of the recorded signal. However, the areas under the curves (AUC) were compared to determine the differences between the time waveforms obtained. The entire recorded measurement cycle was separated into individual gestures, and for each of them (for each component: *a_x_*_,_ *a_y_*, *a_z_*, ω*_x_*, ω*_y_*, and ω*_z_* from 16 sensors), the AUC was calculated.

### 5.2. Statistical Analysis

The statistical analysis of the results was performed using Statistica 13. The distribution of variables in the individual groups was verified with the Shapiro–Wilk W test and graphically assessed. The unpaired t-test and, in justified cases, its non-parametric counterpart, the Mann–Whitney U test, was used to compare the results (AUC) of two groups, i.e., those obtained from complete data and after applying the transmission control algorithm. Although the measurement cycle consisted of a series of gestures repeated three times, they were not treated as dependent samples. The aim was not to check the repeatability of the gestures performed, but to verify the impact of the applied transmission control algorithm on the shape of the obtained measurement signals. For this reason, each repetition of a specific gesture was treated as independent. Each group (individually for each of the six gestures) was verified with the two-sided Grubbs test for outliers. However, none of the values indicated by the test was removed, as the deviation in the values did not result from a measurement error, but from a specific arrangement of sensors on the hand when performing a specific gesture. For example, in the case of gesture 2 and sensor 15 or 16, the changes in values in some components may be much smaller than in the case of other sensors, due to the arrangement of these sensors. The calculated AUC value may deviate from the other values, but should not be treated as a measurement error and removed from the data series.

Table 6 shows the probabilities obtained for the comparison of the AUCs calculated from the signals obtained from the complete data and after applying the data transmission control algorithm. In each of the analysed cases (for each of the six gestures performed), the differences in the AUC turned out to be statistically insignificant (*p* > 0.05).

Table 7 and Table 8 present descriptive statistics (mean, standard deviation, minimum value, maximum value, median, p_25_—lower quartile, p_75_—upper quartile, p_75_–p_25_—interquartile range) obtained from the percentage differences between the AUCs calculated from the complete data and after applying the data transmission control algorithm.

The maximum difference between the AUCs for the accelerometer data was obtained for gesture 3 (Figure 12c) and was about 5–7%, but it was the only case in the entire series of data, none of which exceeded 4.7%. In the case of the gyroscope, also for gesture 3, in one case (only for the z component) the difference in the AUCs exceeded 5%. The mean difference in the AUCs from the entire measurement cycle, both for the data obtained from the accelerometer and the gyroscope, was about 1%. Due to the separation of individual components and the imperfect reproducibility of gestures in the next three series of repetitions, the data are varied, therefore the standard deviations (std) are comparable to the calculated mean values.

## 6. Conclusions

Modern wearable sensor systems require the processing and transmission of more and more data. Energy and teletransmission aspects should be taken into account when designing such solutions. The necessity to transmit large amounts of data, as in the case of the presented sensor system for hand motion acquisition, is related to the load on the radio module and external teletransmission infrastructure. The process of radio data transmission is also a significant energy burden for modern wearable battery-powered sensor systems.

The article presents the algorithm that was implemented in the sensor system and then tested. This algorithm controls data transmission via the Wi-Fi module. Depending on the dynamics of hand motion, the algorithm changes the number of transmitted frames and the length of these frames, which allows to reduce the energy demand of the radio module, but also significantly reduces the load on the Wi-Fi network. This data transmission procedure does not degrade the image quality of the monitored signal. The conducted research shows that new algorithms for data processing and transmission used in sensor systems allow for the improvement of energy (battery operation time) and teletransmission parameters (lower network load). The developed adaptive algorithm can be used to monitor motion with different dynamics, as in the case of gestures, where there is a movement phase (increased amount of transmitted data) and a rest phase (reduced amount of transmitted data).

## Figures and Tables

**Table 1:** Frequency of sending frames depending on the parameter *d_i_*.

Condition	Frequency of Sending Frames *f_T_*
$d_{i}$ ≤ *TH*1	5 Hz
*TH*1 ≤ $d_{i}$ ≤ *TH*2	15 Hz
*TH*2 ≤ $d_{i}$ ≤ *TH*3	30 Hz
$d_{i}$ ≥ *TH*3	60 Hz

**Table 2:** Configurations of defined measurement cases.

Case Number	Number of Sensors for Which Data Are Transmitted with a Fixed Frequency			
	5 Hz	15 Hz	30 Hz	60 Hz
P1	16	0	0	0
P2	14	2	0	0
P3	14	0	2	0
P4	14	0	0	2
P5	0	14	2	0
P6	0	0	14	2
P7	2	0	0	14
P8	13	3	0	0
P9	13	0	3	0
P10	13	0	0	3
P11	0	13	3	0
P12	0	0	13	3
P13	3	0	0	13
P14	8	8	0	0
P15	8	0	8	0
P16	8	0	0	8
P17	0	8	8	0
P18	0	0	8	8
P19	15	1	0	0
P20	15	0	1	0
P21	15	0	0	1
P22	14	1	1	0
P23	13	1	1	1
P24	0	16	0	0
P25	0	0	16	0
P26	0	0	0	16

**Table 3:** WSS current consumption depending on the defined measurement case.

Cases	RMS Current (mA) Mean ± Std
P1	200.97
P2, P8, P14, P19, P24	241.99 ± 0.48
P3, P4, P5, P6, P7, P9, P10, P11, P12, P13, P15, P16, P17, P18, P20, P21, P22, P23, P25, P26	250.82 ± 0.55

**Table 4:** Differences in the radio band occupancy depending on the frame transmission frequency.

Transmission Frequency	5 Hz	15 Hz	30 Hz	60 Hz
**Difference (%)**	−70.23%	−38.05%	−23.84%	Reference

**Table 5:** Differences in data rate depending on the defined measurement case.

Case Number	Mean (kB/s)		Difference (%)	
	Ethernet	Wi-Fi	Ethernet	Wi-Fi
P1	4.50	3.80	−91.60%	−91.52%
P2	6.20	5.49	−88.43%	−87.76%
P3	8.82	9.36	−83.54%	−79.11%
P4	14.05	14.70	−73.79%	−67.22%
P5	15.98	14.44	−70.18%	−67.79%
P6	32.05	29.67	−40.20%	−33.84%
P7	48.98	44.23	−8.62%	−1.36%
P8	6.71	6.44	−87.49%	−85.64%
P9	10.10	9.32	−81.16%	−79.20%
P10	16.86	17.44	−68.54%	−61.11%
P11	16.80	15.46	−68.65%	−65.51%
P12	33.59	30.46	−37.33%	−32.07%
P13	46.66	40.49	−12.95%	−9.69%
P14	9.29	8.04	−82.66%	−82.07%
P15	16.56	15.10	−69.11%	−66.33%
P16	31.02	28.71	−42.12%	−35.96%
P17	20.63	17.00	−61.51%	−62.09%
P18	41.26	37.11	−23.01%	−17.23%
P19	5.69	5.69	−89.39%	−87.30%
P20	7.58	8.34	−85.86%	−81.39%
P21	11.22	12.64	−79.06%	−71.80%
P22	8.04	8.30	−84.99%	−81.50%
P23	13.01	13.09	−75.72%	−70.81%
P24	13.41	11.43	−74.98%	−74.51%
P25	26.77	22.66	−50.04%	−49.46%
P26	53.60	44.84	Reference	

**Table 6:** Probabilities obtained from testing the significance of differences between the AUCs calculated based on the complete data and after applying the algorithm.

Gesture	*p*					
	Accelerometer			Gyroscope		
	ax	ay	az	ωx	ωy	ωz
1	0.9962	0.8980	0.9737	0.9620	0.9679	0.9737
2	0.9737	0.8806	0.8633	0.9753	0.9971	0.8806
3	0.8922	0.9679	0.8806	0.9736	0.9212	0.8748
4	0.5902	0.9854	0.9503	0.8748	0.9800	0.9704
5	0.9854	0.8922	0.8748	0.9912	0.8633	0.9974
6	0.8232	0.8061	0.8690	0.9622	0.9096	0.9888

**Table 7:** Descriptive statistics obtained based on the accelerometer data.

Gesture	Component	Mean ± Std	Min	Max	Median	p_25_	p_75_	p_75_–p_25_
Complete cycle	a_x_	1.21 ± 1.07%	0.00%	6.86%	0.88%	0.45%	1.64%	1.19%
	a_y_	0.96 ± 0.87%	0.00%	5.12%	0.64%	0.26%	1.46%	1.20%
	a_z_	0.92 ± 0.79%	0.00%	6.89%	0.78%	0.35%	1.29%	0.95%
1	a_x_	0.85 ± 0.78%	0.02%	3.35%	0.61%	0.29%	1.17%	0.88%
	a_y_	0.98 ± 0.89%	0.00%	3.52%	0.60%	0.35%	1.52%	1.17%
	a_z_	0.95 ± 0.81%	0.00%	3.23%	0.86%	0.29%	1.32%	1.03%
2	a_x_	0.97 ± 0.96%	0.02%	4.58%	0.73%	0.31%	1.17%	0.87%
	a_y_	0.51 ± 0.75%	0.01%	4.39%	0.23%	0.12%	0.57%	0.45%
	a_z_	0.61 ± 0.48%	0.04%	1.80%	0.43%	0.21%	0.94%	0.73%
3	a_x_	1.42 ± 1.25%	0.01%	6.86%	1.16%	0.68%	1.68%	1.00%
	a_y_	1.05 ± 0.92%	0.00%	5.12%	0.77%	0.48%	1.39%	0.91%
	a_z_	0.97 ± 1.21%	0.00%	6.89%	0.69%	0.31%	1.08%	0.77%
4	a_x_	2.29 ± 1.20%	0.30%	4.28%	2.35%	1.08%	3.41%	1.20%
	a_y_	1.10 ± 0.88%	0.01%	3.47%	1.00%	0.30%	1.76%	1.46%
	a_z_	1.03 ± 0.63%	0.00%	2.46%	0.94%	0.61%	1.33%	0.72%
5	a_x_	0.74 ± 0.53%	0.04%	2.31%	0.62%	0.38%	0.96%	0.58%
	a_y_	1.05 ± 0.99%	0.04%	3.70%	0.72%	0.25%	1.77%	1.52%
	a_z_	1.10 ± 0.76%	0.12%	3.06%	0.88%	0.49%	1.55%	1.06%
6	a_x_	0.97 ± 0.67%	0.00%	2.43%	0.85%	0.50%	1.37%	0.86%
	a_y_	1.01 ± 0.66%	0.08%	2.26%	0.84%	0.55%	1.58%	1.04%
	a_z_	0.84 ± 0.56%	0.01%	2.00%	0.71%	0.38%	1.29%	0.91%

**Table 8:** Descriptive statistics obtained based on the gyroscope data.

Gesture	Component	Mean ± Std	Min	Max	Median	p_25_	p_75_	p_75_–p_25_
Complete cycle	ω_x_	0.85 ± 0.70%	0.00%	3.49%	0.66%	0.30%	1.19%	0.89%
	ω_y_	0.64 ± 0.73%	0.00%	4.84%	0.39%	0.19%	0.82%	0.63%
	ω_z_	0.73 ± 0.83%	0.00%	6.45%	0.45%	0.22%	0.89%	0.67%
1	ω_x_	1.01 ± 0.75%	0.00%	3.49%	0.90%	0.55%	1.30%	0.75%
	ω_y_	0.82 ± 0.89%	0.01%	4.23%	0.53%	0.19%	1.06%	0.87%
	ω_z_	1.12 ± 1.04%	0.01%	5.00%	1.04%	0.51%	1.76%	1.25%
2	ω_x_	0.54 ± 0.48%	0.00%	2.46%	0.35%	0.20%	0.75%	0.55%
	ω_y_	0.52 ± 0.69%	0.01%	4.11%	0.32%	0.14%	0.54%	0.40%
	ω_z_	0.60 ± 0.61%	0.02%	2.87%	0.39%	0.19%	0.91%	0.72%
3	ω_x_	0.98 ± 0.76%	0.00%	3.15%	0.70%	0.37%	1.52%	1.15%
	ω_y_	0.85 ± 1.00%	0.03%	4.84%	0.45%	0.24%	1.11%	0.87%
	ω_z_	1.27 ± 1.21%	0.04%	6.45%	0.93%	0.35%	1.74%	1.39%
4	ω_x_	0.71 ± 0.63%	0.00%	2.64%	0.50%	0.27%	1.09%	0.83%
	ω_y_	0.36 ± 0.33%	0.00%	1.80%	0.33%	0.14%	0.47%	0.33%
	ω_z_	0.42 ± 0.47%	0.01%	2.86%	0.32%	0.17%	0.33%	0.33%
5	ω_x_	0.94 ± 0.62%	0.05%	2.41%	0.71%	0.45%	1.35%	0.90%
	ω_y_	0.70 ± 0.68%	0.03%	3.91%	0.49%	0.22%	1.06%	0.83%
	ω_z_	0.39 ± 0.32%	0.02%	1.94%	0.33%	0.16%	0.52%	0.36%
6	ω_x_	0.90 ± 0.79%	0.01%	2.71%	0.93%	0.14%	1.19%	1.05%
	ω_y_	0.57 ± 0.51%	0.01%	2.31%	0.38%	0.23%	0.78%	0.56%
	ω_z_	0.48 ± 0.32%	0.01%	1.40%	0.45%	0.23%	0.63%	0.40%

**Figure A1:**
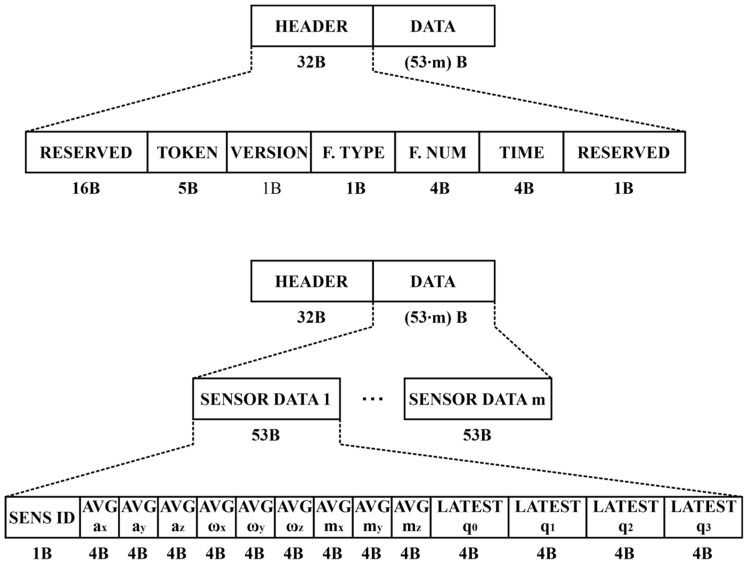
Structure of the WSS data frame.

## Data Availability

Not applicable.

## Appendix A. Structure of the Teletransmission Frame

Figure A1 shows the data frame format. The HEADER block contains tags to identify the device type (TOKEN), its version (VERSION), and the frame type (F.TYPE). The header block further includes the frame number (F.NUM) and the current time (TIME). Elements marked as RESERVED constitute additional data stores that can be used in more complex applications.

The data block (DATA) consists of m sensor data blocks (SENSOR DATA 1,…, SENSOR DATA m), where m ∈ {1, 2…,16}. Only the data from sensors selected for transmission by the algorithm are added to the data section. The sensor data block consists of the sensor number (SENS ID), averaged values of the coordinates of the vectors $a_{i}$ (AVG a_x_, AVG a_y_, AVG a_z_), $\omega_{i}$ (AVG ω_x_, AVG ω_y_, AVG ω_z_), $m_{i}$ (AVG m_x_, AVG m_y_, AVG m_z_), and the current quaternion $q_{i}=\left[q_{0},q_{1},\;q_{2},q_{3}\right]$ (LATEST q_0_, LATEST q_1_, LATEST q_2_, LATEST q_3_). Both the averaged values of the vector coordinates and the elements of the current quaternion are single-precision four-byte floating-point numbers (floats). The total length of a single sensor data block is 53 B as shown in Figure A1.
